# Supplementary material for: Single-cell ATAC and RNA sequencing reveal pre-existing and persistent cells associated with prostate cancer relapse
Source: Nat Commun. 2021 Sep 6;12:5307. doi: 10.1038/s41467-021-25624-1 (PMC8421417; doi:10.1038/s41467-021-25624-1)
Supplement: Supplementary file 1 — Supplementary Information [file 41467_2021_25624_MOESM1_ESM.pdf]

Single-cell ATAC and RNA sequencing reveal pre-existing and persistent cells associated with prostate cancer relapse

Taavitsainen S, et al.

Supplementary Information

Supplementary Figures

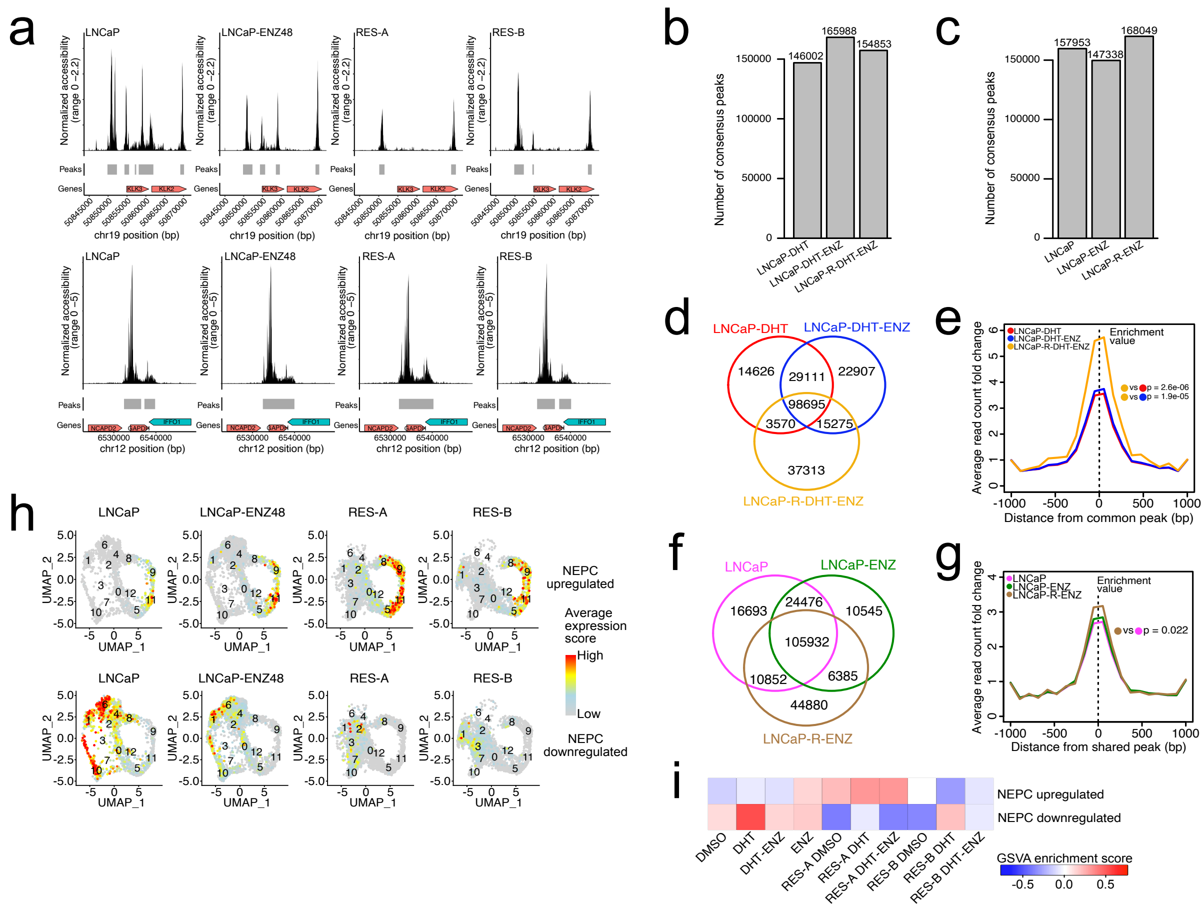

Supplementary Fig. 1 (Relative to Figure 1). (a) Normalized chromatin accessibility for the LNCaP, LNCaP-ENZ48, RES-A, and RES-B samples at the *KLK2*/*KLK3* locus and the *GAPDH* locus. The y-axis depicts the average frequency of DNA fragments sequenced from the regions. The locations of peaks in each sample are shown as grey boxes. **(b-c)** Barplots of consensus formaldehyde-assisted isolation of regulatory elements (FAIRE)-seq peaks in LNCaP samples treated with androgens or in castrate conditions (three biological replicates per condition). **(d)** Venn diagram of FAIRE-seq peaks shared or unique to LNCaP samples in the presence of androgens (dihydrotestosterone, DHT). The enzalutamide (ENZ)-resistant (R) sample had a higher proportion of unique open chromatin sites compared to the LNCaP parental (24% vs 12% unique open sites, $p < 2.2e-16$, chi-square test). **(e)** Normalized average FAIRE-seq read distribution within a 2kb interval around FAIRE-seq sites shared by LNCaP samples in the presence of androgens. Sample comparisons of enrichment values at the middle of the distribution are indicated using colored dots and the two-sided t-test p-values are shown within the plots. **(f)** Venn diagram of FAIRE-seq peaks shared or unique to LNCaP samples in androgen-deprived conditions. The LNCaP ENZ-resistant sample was found to have a higher proportion of unique open chromatin sites compared to the LNCaP parental (27% vs 9% unique open sites, $p < 2.2e-16$, chi-square test). **(g)** Normalized average FAIRE-seq read distribution within a 2kb interval around FAIRE-seq sites shared by LNCaP samples in androgen-deprived conditions. The sample comparison of enrichment values at the middle of the distribution is indicated using colored dots and the two-sided t-test p-value is shown within the plot. **(h)** Feature plot showing the average expression score

of each cell in the four LNCaP scRNA-seq samples for the neuroendocrine prostate cancer (NEPC) up- and downregulated gene sets. Red indicates high expression and grey indicates low expression. The clusters are labeled with numbers in each plot. **(i)** Gene set variation analysis (GSVA) enrichment scores for NEPC up- and downregulated genes in bulk RNA sequencing of LNCaP exposed to various treatments.

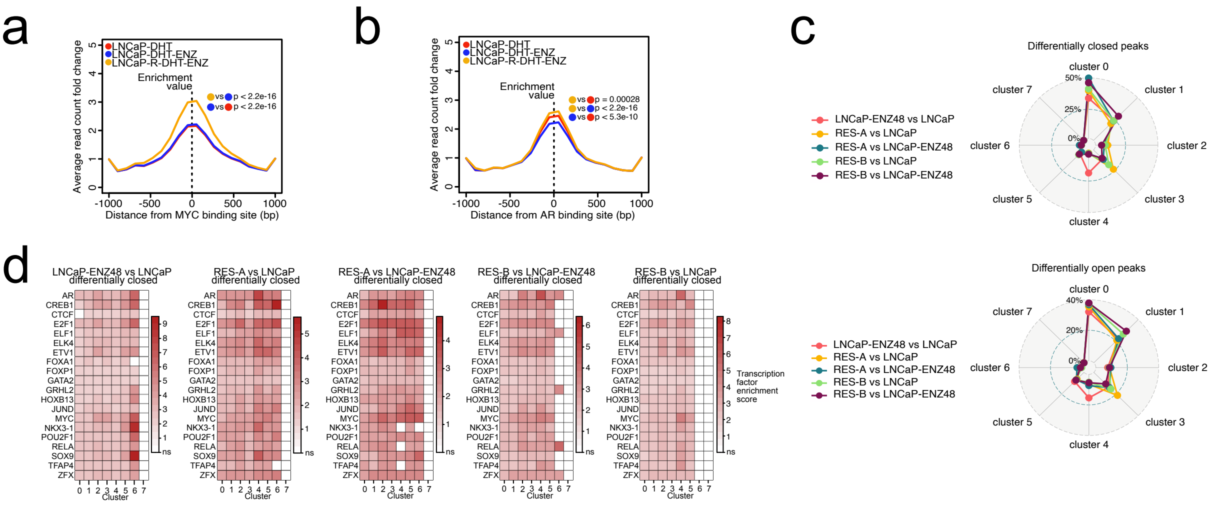

Supplementary Fig. 2 (Relative to Figure 2). (a-b) Normalized average formaldehyde-assisted isolation of regulatory elements (FAIRE)-seq read distribution of LNCaP samples in androgen-exposed conditions within a 2kb window around **(a)** MYC binding sites and **(b)** AR binding sites. Sample comparisons of enrichment values at the middle of the distribution are indicated using colored dots and the two-sided t-test p-values are shown within the plots. **(c)** Radar plots of open and closed differentially accessible chromatin regions (DARs) in pairwise sample comparisons, shown as a percentage of the total open or closed DARs between sample conditions. **(d)** Prostate cancer-associated transcription factor motif enrichments in closed DARs in pairwise sample comparisons. Enrichments with a Benjamini-Hochberg method adjusted hypergeometric test p-value < 0.05 are shown in shades of red, while non-significant (ns) enrichment are shown in white. ENZ = enzalutamide, DHT = dihydrotestosterone.

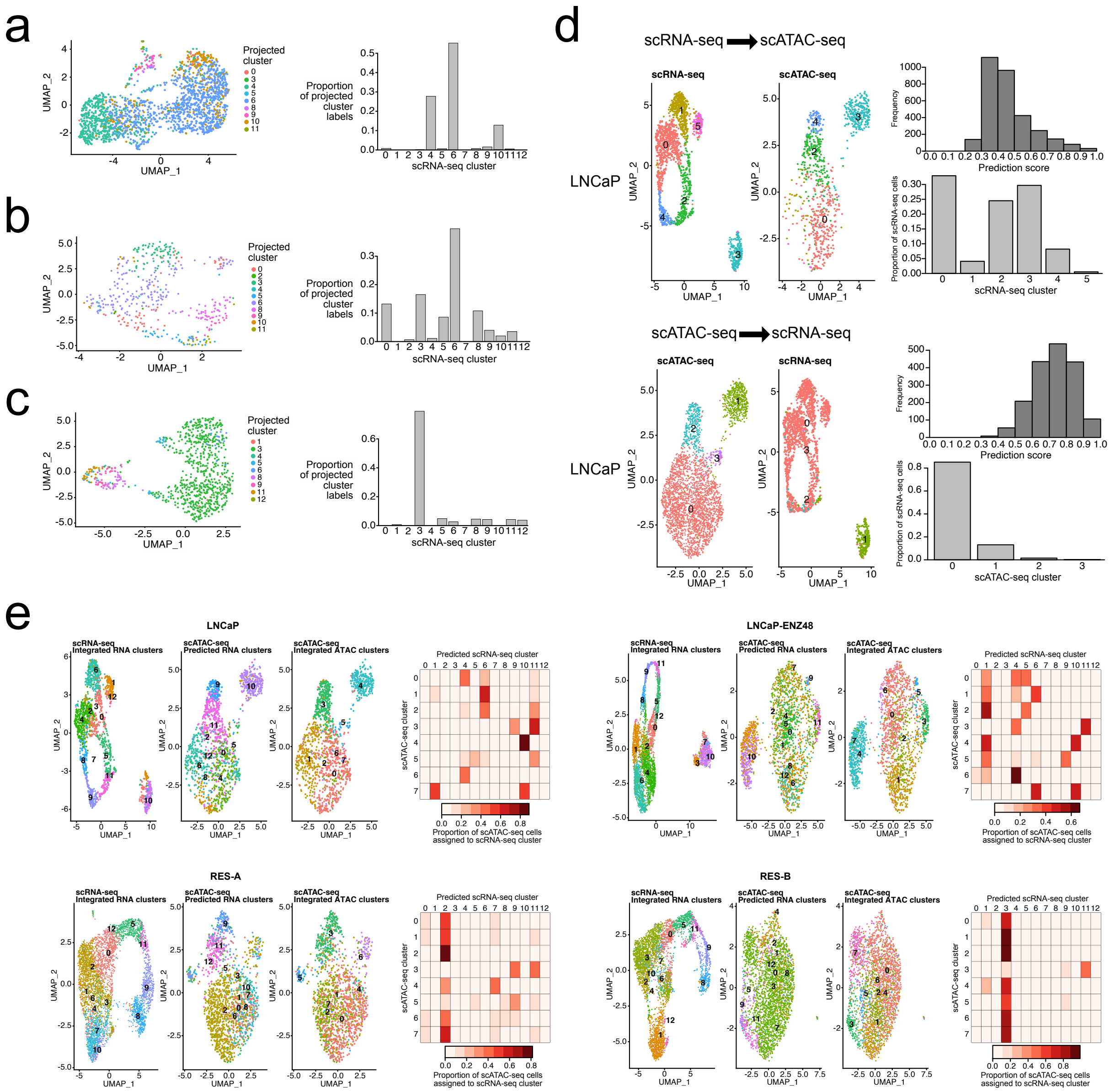

Supplementary Fig. 3 (Relative to Figure 3). (a) Cluster label transfer from integrated clustering of the single-cell (sc) RNA-seq data to an LNCaP sample treated with DMSO from an external dataset. Each cell is colored according to the scRNA-seq cluster that it is predicted to belong to. The barplot shows the proportion of the projected cluster labels for each scRNA-seq cluster. **(b)** Cluster label transfer from integrated clustering of the scRNA-seq data to an enzalutamide (ENZ)-resistant LNCaP sample treated with ENZ for 9 months (RES-C). Each cell is colored according to the scRNA-seq cluster that it is predicted to belong to. The barplot shows the proportion of the projected cluster labels for each scRNA-seq cluster. **(c)** Cluster label transfer from integrated clustering of the scRNA-seq data to a scRNA-seq LNCaP sample treated with ENZ for 168 hours (LNCaP-ENZ168). For the LNCaP-ENZ168 sample, each cell is colored according to the scRNA-seq cluster that it is predicted to belong to. The barplot shows the proportion of the projected cluster labels for each scRNA-seq cluster. **(d)** Sample-wise transfer of cluster labels from scRNA-seq to scATAC-seq, and from scATAC-seq to scRNA-seq, exemplified in parental LNCaP. For each cluster label transfer direction, the prediction scores of the assigned cluster labels for each cell in the query are plotted as a histogram, and the proportion of query cells assigned to

each cluster from the reference is shown as a barplot. In the top figure set, the scRNA-seq clusters are used as the reference and projected onto the scATAC-seq cells. In the scATAC-seq UMAP, cells that could be assigned a cluster label from the scRNA-seq with a prediction score of 0.5 or higher are colored according to their predicted scRNA-seq cluster assignment. In the bottom figure set, the cluster label transfer process is performed by using the scATAC-seq clusters as the reference and projecting the cluster labels onto the scRNA-seq cells. **(e)** Sample-wise transfer of cluster labels from scRNA-seq to scATAC-seq for the integrated scRNA-seq clusters as shown in **Fig. 3a**. For each sample condition, the scRNA-seq sample was clustered individually and the cells labeled according to their integrated clusters. These clusters were then queried in the scATAC-seq cells and those with label transfer prediction scores > 0.3 were labeled according to their predicted scRNA-seq cluster. The same scATAC-seq cells were finally labeled according to their integrated scATAC-seq cluster to visualize the matching cell states between the data types. For each sample condition, a matrix is shown to depict the proportion of cells from each scATAC-seq cluster in the sample condition assigned to each scRNA-seq cluster. The proportions were calculated for each scATAC-seq cluster, with the total as the number of cells from the scATAC-seq that could be confidently assigned to an scRNA-seq cluster (prediction score > 0.3). UMAP = Uniform Manifold Approximation and Projection.

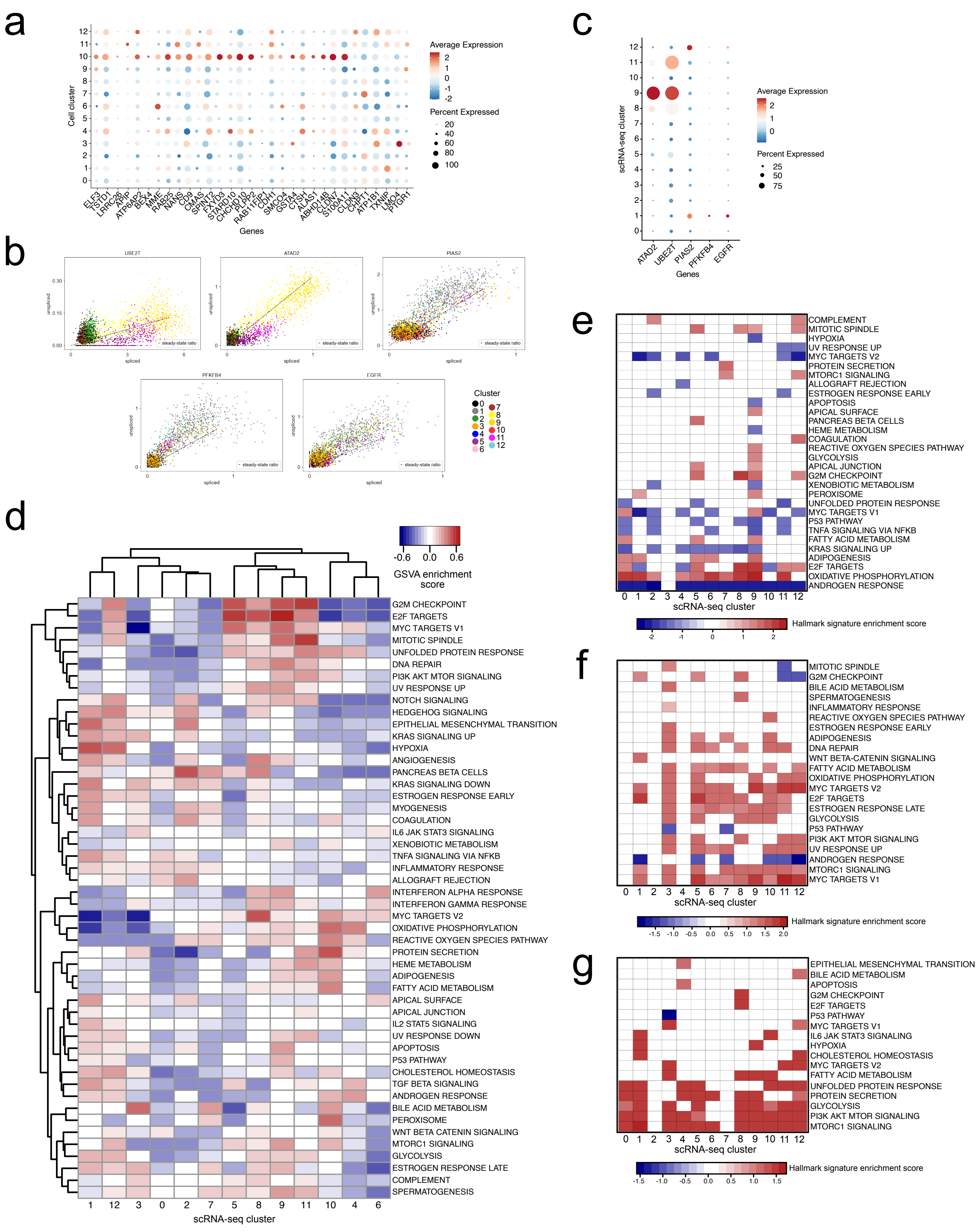

Supplementary Fig. 4 (Relative to Figure 4). (a) Dot plot of average gene expression of the PROSGenesis signature genes in the single-cell (sc) RNA-seq clusters. The plot includes only the signature genes from Karthaus et al.[1] expressed by over 40% of cells in one or more clusters. **(b)** Examples of marker genes for RNA velocity across clusters identified from scRNA-seq for RES-A and RES-B. The abundance of spliced versus unspliced mRNA is shown for each gene in each cell, with the black dashed line indicating equal amounts of spliced and unspliced mRNA. Clusters of cells identified from scRNA-seq are shown in different colors. **(c)** Expression of RNA velocity marker genes in the clusters

identified from scRNA-seq shown as a dot plot. **(d)** Molecular Signatures Database **(**MSigDB) hallmark gene set enrichments using gene set variation analysis (GSVA) based on the average gene expression profile of each scRNA-seq cluster presented as a clustered heatmap. The heatmap colors reflect the GSVA scores of each hallmark signature in each scRNA-seq cluster. **(e-g)** Gene set enrichment analysis (GSEA) of MSigDB hallmark gene sets for scRNA-seq sample comparisons. Enrichments are shown for changes in gene expression between **(e)** LNCaP-ENZ48 and LNCaP, **(f)** RES-A and LNCaP, and **(g)** RES-B and LNCaP. The heatmap colors correspond to normalized GSEA enrichment scores with Benjamini-Hochberg adjusted p-values < 0.05. Hallmark gene sets with adjusted p-values > 0.05 are shown in white.

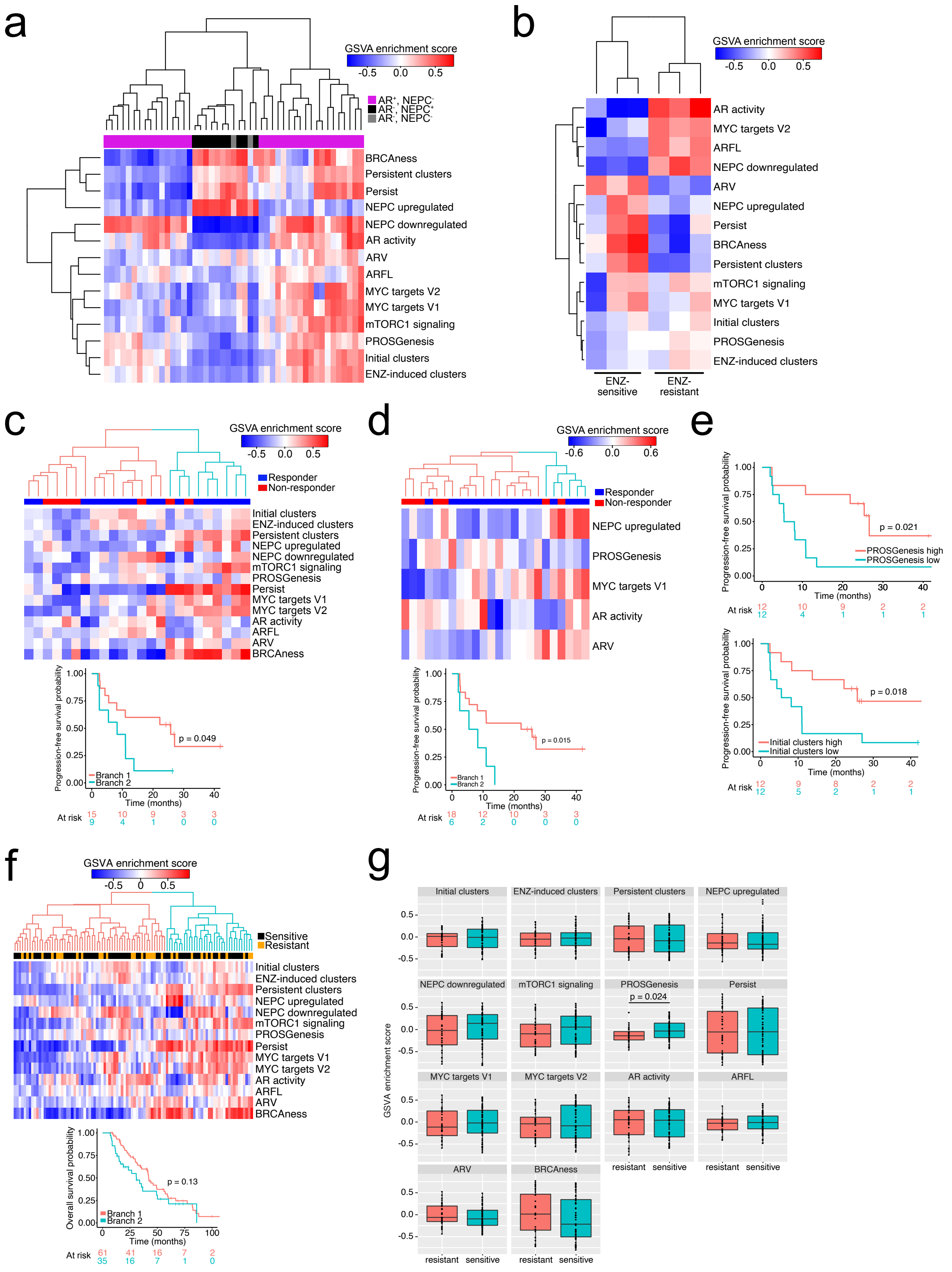

Supplementary Fig. 5 (Relative to Figure 5). (a) Gene set variation analysis (GSVA) enrichment score heatmap for tumors of varying AR and neuroendocrine prostate cancer (NEPC) status[2,3] based on the single-cell gene signatures. **(b)** GSVA enrichment score heatmap for enzalutamide (ENZ)-sensitive and ENZ-resistant xenografts[4] based on the single-cell gene signatures. **(c)** GSVA enrichment scores for patients from Alumkal et al.[5] for

all single-cell gene signatures, with hierarchical clustering of the patients into two groups based on the scores indicated in pink and cyan in the column dendrogram. The Kaplan-Meier curve shows the patients stratified into two groups determined by the clustering. The two-sided log-rank p-value is indicated above the curve. **(d)** GSVA enrichment scores for patients from Alumkal et al. for the five gene signatures selected via stepwise variable selection to form the best Cox proportional hazards model. The Kaplan-Meier curve shows the two groups of patients identified via hierarchical clustering of the GSVA scores. The two-sided log-rank p-value is indicated above the curve. **(e)** Kaplan-Meier curves for patients from Alumkal et al. stratified into two groups based on median GSVA score for the PROSGenesis and initial cluster signatures. The two-sided log-rank p-value is indicated above the curves. **(f)** Single-cell-derived gene sets cannot be used to identify patients with shorter overall survival in the SU2C West Coast Dream Team dataset from Quigley et al.[6] A heatmap of GSVA enrichment scores is shown for the patients from Quigley et al. Hierarchical clustering of the patients into two groups is shown by the pink and cyan colors in the column dendrogram. This patient grouping was used to stratify patients for the overall survival Kaplan-Meier curve. The two-sided log-rank p-value is indicated above the curves. **(g)** Boxplots of GSVA enrichment scores for ENZ-resistant and -sensitive patients from Quigley et al. Pink boxplots indicate the GSVA scores of ENZ-resistant samples and cyan boxplots show the GSVA scores of ENZ-sensitive samples. Differences in GSVA scores for signatures between ENZ-resistant (n=32 biologically independent samples) and ENZ-sensitive (n=64 biologically independent samples) samples were assessed using the two-sided Wilcoxon rank sum test, with the PROSGenesis signature showing a significant difference (p = 0.024). The boxplots show the 25th percentile, median, and 75th percentile, with the whiskers indicating the minimum and maximum values within the 1.5x interquartile range.

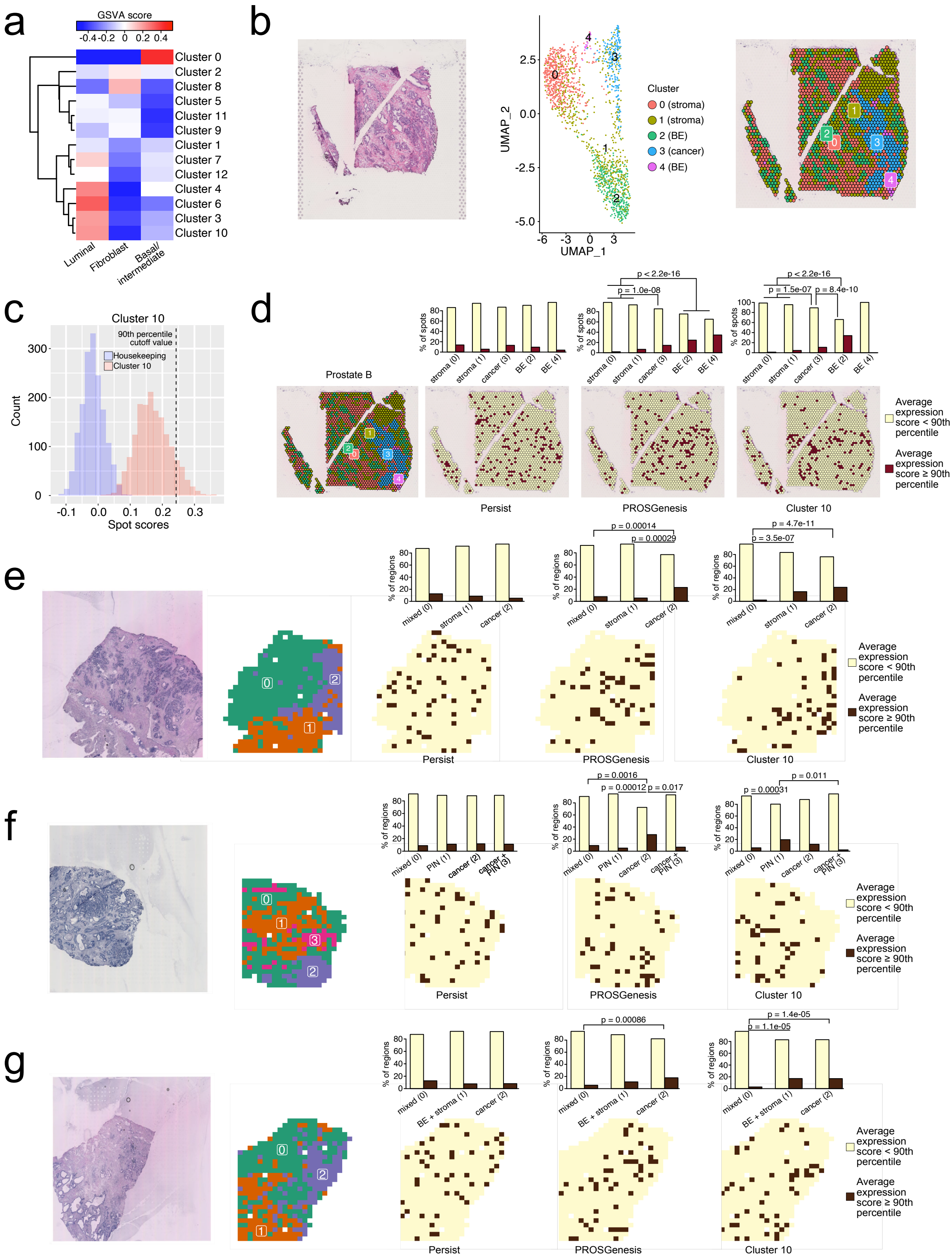

Supplementary Fig. 6 (Relative to Figure 6). (a) Gene set variation analysis (GSVA) enrichment scores for LNCaP single-cell RNA-seq individual cluster signatures in luminal, basal/intermediate, and fibroblast cells from Chen et al.[7] GSVA enrichment scores were generated from the average expression profile of each cell type. Luminal and basal/intermediate cells had higher expression of all LNCaP model-derived cell clusters

compared to fibroblasts (average score -0.07 vs -0.20, p = 0.047, two-sided t-test). Luminal cells also showed higher expression of the genes characterizing the initial scRNA-seq clusters (clusters 4, 6, and 10) than basal/intermediate cells (average score 0.23 vs -0.12, p = 0.020, two-sided t-test) and compared to fibroblasts (average score 0.23 vs -0.39, p = 0.00015, two-sided t-test). **(b-d)** Spatial transcriptomics (ST) from a prostate cancer tissue section, Prostate B. **(b)** The left panel shows the hematoxylin and eosin (H&E) staining of the tissue section. In the middle, the Uniform Manifold Approximation and Projection (UMAP) visualization shows the clusters of spots on the ST slide. Each cluster is labeled according to its histological tissue type, with clusters 0 and 1 corresponding to stroma, clusters 2 and 4 corresponding to benign epithelium (BE), and cluster 3 corresponding to the prostate adenocarcinoma. The right panel shows the UMAP clusters of spots overlaid on the H&E slide. **(c)** Sensitivity analysis of Cluster 10 gene signatures scores in ST against the score distribution of a control housekeeping gene signature (see **Methods**). **(d)** The leftmost panel shows the UMAP clusters of spots overlaid on the H&E slide. Each spot was scored according to its expression of genes in the Persist, PROSGenesis, and cluster 10 signatures. For each signature, spots scoring at or above the 90th percentile (high) are colored in red, while spots scoring below the 90th percentile (low) are colored in yellow. The barplots indicate the percentage of spots in each cluster scoring high or low for each signature. The bars are labeled with the cluster histology and the cluster number in parentheses, with BE referring to benign epithelium. Differences in proportions of high scoring spots were tested between clusters with the chi-square test. **(e-g)** ST from prostate cancer tissue sections **(e)** 3.3, **(f)** 1.2, and **(g)** 2.4 from Berglund et al.[8] For each section, the left panel shows the section H&E image (modified from Berglund et al.) adjacent to the UMAP clusters of spots determined from RNA data overlaid on the H&E slide. Each spot was scored according to its expression of genes in the Persist, PROSGenesis, and cluster 10 signatures. For each signature, spots scoring at or above the 90th percentile (high) are colored in brown, while spots scoring below the 90th percentile (low) are colored in yellow. The barplots indicate the percentage of spots in each cluster scoring high or low for each signature. The bars are labeled with the cluster histology and the cluster number in parentheses. In the labels, mixed refers to the cluster containing spots in both cancerous and benign regions, BE stands for benign epithelium, and PIN stands for prostatic intraepithelial neoplasia. Differences in proportions of high scoring spots between clusters were tested with the chi-square test.

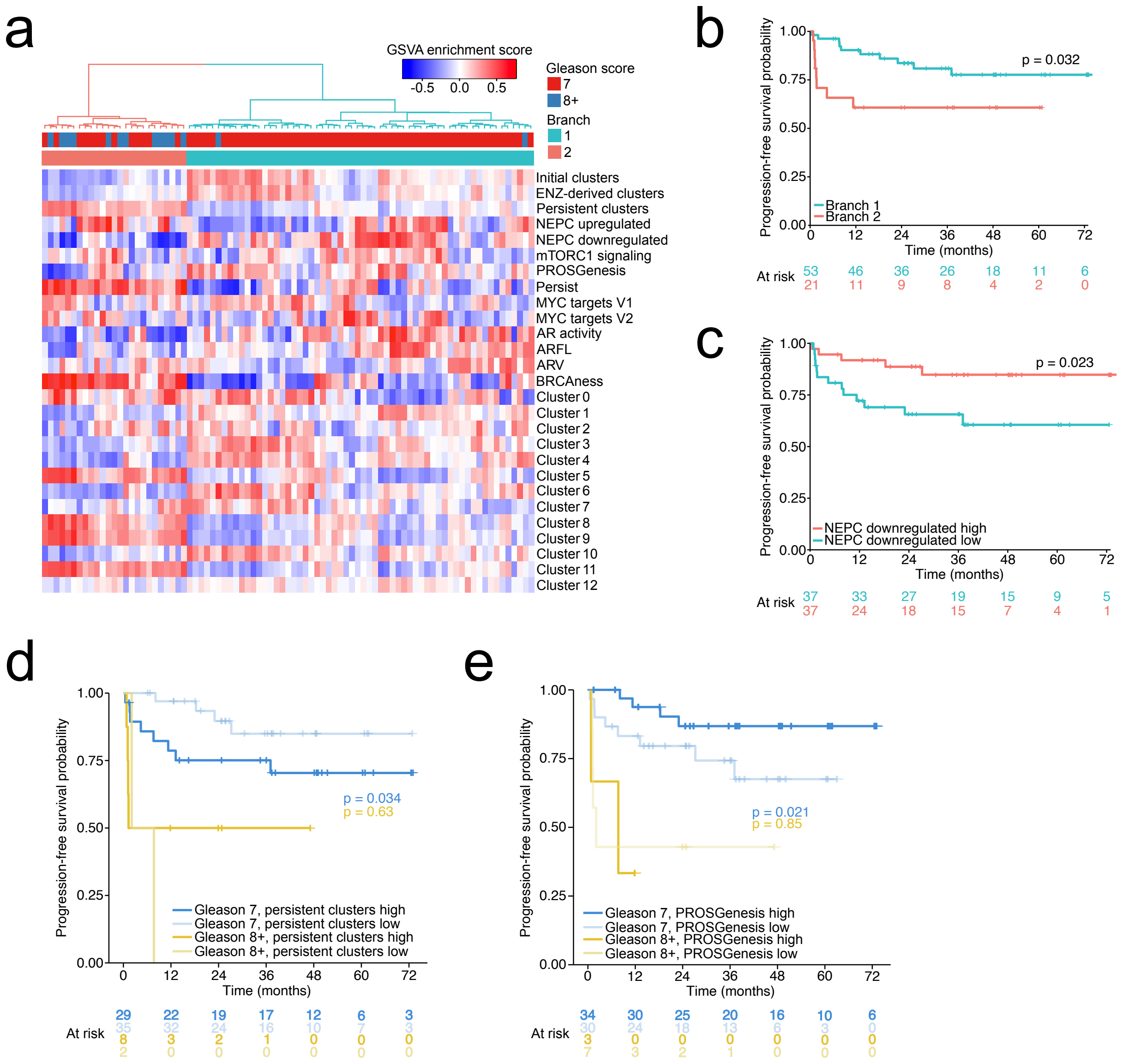

Supplementary Fig. 7 (Relative to Figure 7). (a) Heatmap of gene set variation analysis (GSVA) enrichment scores for all single-cell-derived gene signatures in the ICGC early onset prostate cancer (EOPC)[9] cohort, including the marker gene set for each single-cell RNA-seq cluster. Hierarchical clustering of the GSVA scores was used to separate the samples into two groups, labeled as Branch 1 and Branch 2. **(b)** Kaplan-Meier survival curve for ICGC-EOPC patients stratified into two groups as indicated in Panel **a**. **(c)** Kaplan-Meier survival curves for ICGC-EOPC patients stratified into two groups based on median GSVA score for the neuroendocrine prostate cancer (NEPC) downregulated gene signature. **(d-e)** Kaplan-Meier curves for ICGC-EOPC patients stratified into four groups based on Gleason score and median GSVA score for the persistent cluster signature or the PROSGenesis signature. Two-sided log-rank p-values are shown within the plots for all Kaplan-Meier survival curves.

Supplementary Tables

Supplementary Table 1. Details of datasets utilized in the study. For each dataset, the table indicates the source of the data (this study or a prior work) and where the data was obtained. A dataset obtained from the authors of a publication is indicated with "Received from authors" in the Identifier column.

Dataset	Source	Identifier
Single-cell RNA- and ATAC-sequencing of LNCaP	This study	GEO: GSE168669
FAIRE-seq of LNCaP	This study	GEO: GSE168669
Spatial transcriptomics data from prostate sections Prostate A and Prostate B	This study	European Genome-Phenome Archive: EGAS00001000526
Single cell RNA-sequencing of LNCaP and VCaP from group of Prof. Gerhardt Attard, UCL	This study	GEO: GSE168733
RNA-sequencing of VCaP models of CRPC and resistance to AR signaling-targeted treatments from group of Prof. Teemu Murtola, Tampere University	This study	GEO: GSE168669
AR and c-MYC binding sites map	Supplementary Reference [10]	GEO: GSE73994
Bulk RNA-sequencing of LNCaP samples	Supplementary Reference [11]	GEO: GSE130534
Xenografts of AR-positive / NE-negative and AR-negative / NE-positive CRPC tumors	Supplementary Reference [2,3]	GEO: GSE124704<br>GEO: GSE126078
LNCaP xenograft model of CRPC	Supplementary Reference [4]	Supplementary File 1 in publication

Patient RNA-sequencing from enzalutamide responders and non-responders	Supplementary Reference [5]	Received from authors
RNA-sequencing from SU2C CRPC patient samples	Supplementary Reference [12]	Received from authors
RNA-sequencing from SU2C West Coast DT patient samples	Supplementary Reference [6]	Received from authors
Single cell RNA-sequencing of LNCaP from group of Dr. Kirsi Ketola, University of Eastern Finland	Unpublished	Received from Ketola lab
scRNA-seq of 13 treatment-naive prostate tumor samples	Supplementary Reference [7]	GEO: GSE141445
Spatial transcriptomics data from prostate tissue sections 1.2, 2.4, and 3.3	Supplementary Reference [8]	Received from authors
TCGA-PRAD RNA-seq	https://portal.gdc.cancer.gov/	https://portal.gdc.cancer.gov/
ICGC-EOPC RNA-seq	Supplementary Reference [9]	Received from authors
Hallmark gene sets from the Molecular Signatures Database (MSigDB) v7.0	Supplementary Reference [13,14]	http://www.gsea-msigdb.org/gsea/msigdb/index.jsp
GTRD database v18.06	Supplementary Reference [15]	https://gtrd.biouml.org/
Housekeeping and Reference Transcript Atlas v1.0	Supplementary Reference [16]	http://www.housekeeping.unicamp.br/

Supplementary Table 2. Details of the software and tools utilized in the study. For each tool, its associated publications (if any) are indicated in the Source column. The Identifier column indicates the download location of the tool.

Resource	Source	Identifier
Cell Ranger (version 3.0.2)	Supplementary Reference [17]	10x Genomics
Cell Ranger ATAC (version 1.1.0)	Supplementary Reference [18]	10x Genomics
Space Ranger (version 1.2.0)	-	10x Genomics
Seurat (version 3.2.0)	Supplementary Reference [19,20]	https://cran.r-project.org/web/packages/Seurat/
sctransform (version 0.3.1)	Supplementary Reference [21]	https://cran.r-project.org/web/packages/sctransform/
fastMNN / batchelor (version 1.2.4)	Supplementary Reference [22]	https://bioconductor.org/packages/release/bioc/html/batchelor.html
MAST (version 1.12.0)	Supplementary Reference [23]	https://www.bioconductor.org/packages/release/bioc/html/MAST.html
GSVA (version 1.34.0)	Supplementary Reference [24]	https://bioconductor.org/packages/release/bioc/html/GSVA.html
fgsea (version 1.14.0)	Supplementary Reference [25]	https://bioconductor.org/packages/release/bioc/html/fgsea.html
scVelo (version 0.2.2)	Supplementary Reference [26]	https://pypi.org/project/scvelo/
cytoTRACE (version 0.3.3)	Supplementary Reference [27]	https://cytotrace.stanford.edu/
Signac (version 0.2.5)	Supplementary Reference [28]	https://github.com/timoast/signac
harmony (version 1.0)	Supplementary Reference [29]	https://github.com/immunogenomics/harmony

ggradar (version 0.2)	-	https://github.com/ricardo-bion/ggradar
TFBSTools (version 1.26.0)	Supplementary Reference [30]	http://bioconductor.org/packages/release/bioc/html/TFBSTools.html
BSgenome.Hsapiens.UCSC.hg38 (version 1.4.1)	-	https://bioconductor.org/packages/release/data/annotation/html/BSgenome.Hsapiens.UCSC.hg38.html
clustree (version 0.4.3)	Supplementary Reference [31]	https://github.com/lazappi/clustree
survival (version 3.2-3)	Supplementary Reference [32]	https://cran.r-project.org/package=survival/
survminer (version 0.4.8)	-	https://cran.r-project.org/we/packages/survminer/
Drop-seq tools (version 2.3.0)	Supplementary Reference [33]	https://github.com/broadinstitute/Drop-seq
bwa (version 0.7.8-r455)	Supplementary Reference [34]	http://bio-bwa.sourceforge.net/
Picard (versions 1.118 and 2.18.22)	-	https://broadinstitute.githu.io/picard/
MACS2 (version 2.1.0)	Supplementary Reference [35]	https://github.com/jsh58/MACS
MSPC (version 4.0.2)	Supplementary Reference [36]	https://genometric.github.io/MSPC/
DiffBind (version 2.14.0)	Supplementary Reference [37]	https://www.bioconductor.org/packages/release/bioc/html/DiffBind.html
featureCounts (version 1.6.2)	Supplementary Reference [38]	http://subread.sourceforge.net/
STAR (versions 2.5.4b and 2.7.3a)	Supplementary Reference [39]	https://github.com/alexdobin/STAR
JASPAR2018 (version 1.1.1)	-	https://bioconductor.org/packages/release/data/annotation/html/JASPAR2018.html
loompy (version 3.0.0)	-	http://loompy.org/

ReactomePA (version 1.30.0)	Supplementary Reference [40]	http://bioconductor.org/packages/release/bioc/html/ReactomePA.html

Supplementary Table 3. Assessment of label transfer prediction score thresholds. Percentage of scATAC-seq cells in each sample label transferred to an integrated cluster from scRNA-seq with different prediction score thresholds.

	**% of scATAC-seq cells label transferred with threshold 0.4**	**% of scATAC-seq cells label transferred with threshold 0.3**	**% of scATAC-seq cells label transferred with threshold 0.2**	**% of scATAC-seq cells label transferred with threshold 0.1**
**LNCaP**	22.50%	44.80%	90.60%	100%
**LNCaP-ENZ48**	29.50%	66.50%	99.00%	100%
**RES-A**	15.10%	49.30%	96.30%	100%
**RES-B**	67.80%	90.70%	99.80%	100%

Supplementary References

1. Karthaus, W. R. *et al.* Regenerative potential of prostate luminal cells revealed by single-cell analysis. *Science* **368**, 497–505 (2020).
2. Lam, H.-M. *et al.* Durable Response of Enzalutamide-resistant Prostate Cancer to Supraphysiological Testosterone Is Associated with a Multifaceted Growth Suppression and Impaired DNA Damage Response Transcriptomic Program in Patient-derived Xenografts. *Eur. Urol.* **77**, 144–155 (2020).
3. Labrecque, M. P. *et al.* Molecular profiling stratifies diverse phenotypes of treatment-refractory metastatic castration-resistant prostate cancer. *J. Clin. Invest.* **129**, 4492–4505 (2019).
4. King, C. J. *et al.* Integrative molecular network analysis identifies emergent enzalutamide resistance mechanisms in prostate cancer. *Oncotarget* **8**, 111084–111095 (2017).
5. Alumkal, J. J. *et al.* Transcriptional profiling identifies an androgen receptor activity-low, stemness program associated with enzalutamide resistance. *Proc. Natl. Acad. Sci. U. S.*

A. **117**, 12315–12323 (2020).

6. Quigley, D. A. *et al.* Genomic Hallmarks and Structural Variation in Metastatic Prostate Cancer. *Cell* **175**, 889 (2018).
7. Chen, S. *et al.* Single-cell analysis reveals transcriptomic remodellings in distinct cell types that contribute to human prostate cancer progression. *Nat. Cell Biol.* **23**, 87–98 (2021).
8. Berglund, E. *et al.* Spatial maps of prostate cancer transcriptomes reveal an unexplored landscape of heterogeneity. *Nature Communications* vol. 9 (2018).
9. Gerhauser, C. *et al.* Molecular Evolution of Early-Onset Prostate Cancer Identifies Molecular Risk Markers and Clinical Trajectories. *Cancer Cell* **34**, 996–1011.e8 (2018).
10. Barfeld, S. J. *et al.* c-Myc Antagonises the Transcriptional Activity of the Androgen Receptor in Prostate Cancer Affecting Key Gene Networks. *EBioMedicine* **18**, 83–93 (2017).
11. Handle, F. *et al.* Drivers of AR indifferent anti-androgen resistance in prostate cancer cells. *Sci. Rep.* **9**, 13786 (2019).
12. Abida, W. *et al.* Genomic correlates of clinical outcome in advanced prostate cancer. *Proc. Natl. Acad. Sci. U. S. A.* **116**, 11428–11436 (2019).
13. Subramanian, A. *et al.* Gene set enrichment analysis: a knowledge-based approach for interpreting genome-wide expression profiles. *Proc. Natl. Acad. Sci. U. S. A.* **102**, 15545–15550 (2005).
14. Liberzon, A. *et al.* The Molecular Signatures Database Hallmark Gene Set Collection. *Cell Systems* vol. 1 417–425 (2015).
15. Kolmykov, S. *et al.* GTRD: an integrated view of transcription regulation. *Nucleic Acids Res.* **49**, D104–D111 (2021).
16. Hounkpe, B. W., Chenou, F., de Lima, F. & De Paula, E. V. HRT Atlas v1.0 database: redefining human and mouse housekeeping genes and candidate reference transcripts by mining massive RNA-seq datasets. *Nucleic Acids Res.* **49**, D947–D955 (2021).
17. Zheng, G. X. Y. *et al.* Massively parallel digital transcriptional profiling of single cells.

Nat. Commun. **8**, 14049 (2017).

18. Satpathy, A. T. *et al.* Massively parallel single-cell chromatin landscapes of human immune cell development and intratumoral T cell exhaustion. *Nat. Biotechnol.* **37**, 925–936 (2019).
19. Butler, A., Hoffman, P., Smibert, P., Papalexi, E. & Satija, R. Integrating single-cell transcriptomic data across different conditions, technologies, and species. *Nat. Biotechnol.* **36**, 411–420 (2018).
20. Stuart, T. *et al.* Comprehensive Integration of Single-Cell Data. *Cell* **177**, 1888–1902.e21 (2019).
21. Hafemeister, C. & Satija, R. Normalization and variance stabilization of single-cell RNA-seq data using regularized negative binomial regression. *Genome Biol.* **20**, 296 (2019).
22. Haghverdi, L., Lun, A. T. L., Morgan, M. D. & Marioni, J. C. Batch effects in single-cell RNA-sequencing data are corrected by matching mutual nearest neighbors. *Nat. Biotechnol.* **36**, 421–427 (2018).
23. Finak, G. *et al.* MAST: a flexible statistical framework for assessing transcriptional changes and characterizing heterogeneity in single-cell RNA sequencing data. *Genome Biol.* **16**, 278 (2015).
24. Hänzelmann, S., Castelo, R. & Guinney, J. GSVA: gene set variation analysis for microarray and RNA-seq data. *BMC Bioinformatics* **14**, 7 (2013).
25. Korotkevich, G., Sukhov, V. & Sergushichev, A. Fast gene set enrichment analysis. doi:10.1101/060012.
26. Bergen, V., Lange, M., Peidli, S., Wolf, F. A. & Theis, F. J. Generalizing RNA velocity to transient cell states through dynamical modeling. *Nat. Biotechnol.* **38**, 1408–1414 (2020).
27. Gulati, G. S. *et al.* Single-cell transcriptional diversity is a hallmark of developmental potential. *Science* **367**, 405–411 (2020).
28. Stuart, T., Srivastava, A., Lareau, C. & Satija, R. Multimodal single-cell chromatin analysis with Signac. *bioRxiv* (2020).

29. Korsunsky, I. *et al.* Fast, sensitive and accurate integration of single-cell data with Harmony. *Nat. Methods* **16**, 1289–1296 (2019).
30. Tan, G. & Lenhard, B. TFBSTools: an R/bioconductor package for transcription factor binding site analysis. *Bioinformatics* vol. 32 1555–1556 (2016).
31. Zappia, L. & Oshlack, A. Clustering trees: a visualization for evaluating clusterings at multiple resolutions. *GigaScience* vol. 7 (2018).
32. Therneau, T. M. & Grambsch, P. M. Modeling Survival Data: Extending the Cox Model. *Statistics for Biology and Health* (2000) doi:10.1007/978-1-4757-3294-8.
33. Macosko, E. Z. *et al.* Highly Parallel Genome-wide Expression Profiling of Individual Cells Using Nanoliter Droplets. *Cell* **161**, 1202–1214 (2015).
34. Li, H. & Durbin, R. Fast and accurate long-read alignment with Burrows-Wheeler transform. *Bioinformatics* **26**, 589–595 (2010).
35. Zhang, Y. *et al.* Model-based analysis of ChIP-Seq (MACS). *Genome Biol.* **9**, R137 (2008).
36. Jalili, V., Matteucci, M., Masseroli, M. & Morelli, M. J. Using combined evidence from replicates to evaluate ChIP-seq peaks. *Bioinformatics* vol. 34 2338–2338 (2018).
37. Stark, R., Brown, G. & Others. DiffBind: differential binding analysis of ChIP-Seq peak data. *R package version* **100**, (2011).
38. Liao, Y., Smyth, G. K. & Shi, W. featureCounts: an efficient general purpose program for assigning sequence reads to genomic features. *Bioinformatics* **30**, 923–930 (2014).
39. Dobin, A. et al. STAR: ultrafast universal RNA-seq aligner. *Bioinformatics* **29**, 15–21 (2013).
40. Yu, G. & He, Q.-Y. ReactomePA: an R/Bioconductor package for reactome pathway analysis and visualization. *Mol. Biosyst.* **12**, 477–479 (2016).
