# Supplementary material for: Single-cell ATAC and RNA sequencing reveal pre-existing and persistent cells associated with prostate cancer relapse
Source: Nat Commun. 2021 Sep 6;12:5307. doi: 10.1038/s41467-021-25624-1 (PMC8421417; doi:10.1038/s41467-021-25624-1)
Supplement: Supplementary file 3 — Description of Additional Supplementary Files [file 41467_2021_25624_MOESM3_ESM.pdf]

**Supplementary Data 1. Single-cell ATAC sequencing.** 1) Set of differentially accessible chromatin regions (DARs) in each scATAC-seq cluster (compared to all other clusters), referred to as marker differentially accessible regions. The regions are annotated with their nearest gene. 2-5) Set of DARs in each scATAC-seq cluster in each sample, compared to all other clusters in the sample. 6-8) Sets of DARs for each pairwise sample comparison (LNCaP-ENZ48 vs LNCaP, RES-A vs LNCaP, and RES-B vs LNCaP). The regions are annotated with their nearest gene. In the tables, the header indicates the scATAC-seq cluster, the differentially accessible region, the average log-fold change in accessibility for the cells in the cluster, the proportion of cells with accessible chromatin in the region in each sample condition or cluster, the likelihood ratio test p-value for the region, and the Bonferroni adjusted p-value for the region.

**Supplementary Data 2. Single-cell RNA sequencing.** 1) Set of genes differentially expressed by each scRNA-seq cluster (compared to all other clusters), referred to as marker differentially expressed genes. 2-4) Sets of differentially expressed genes for each pairwise sample comparison (LNCaP-ENZ48 vs LNCaP, RES-A vs LNCaP, and RES-B vs LNCaP). In each, the table header indicates the scRNA-seq cluster, the differentially expressed gene, the average log-fold change for the cells in the cluster, the proportion of cells expressing the gene in each sample condition, the MAST framework p-value for the gene, and the Bonferroni adjusted p-value for the gene.

**Supplementary Data 3. Signature gene sets derived and used in the study.** Signatures are grouped by 1) individual single-cell cluster marker gene sets, which are genes that define each single-cell RNA sequencing cluster from LNCaP; 2) combined cluster marker gene sets, which are the individual single-cell cluster marker gene sets grouped by cluster type (either initial, ENZ-induced, or persistent); and 3) gene sets representing pathways and processes, including the Persist and PROSGenesis signatures.

**Supplementary Data 4. Quality control thresholds and metrics for the scRNA-seq and scATAC-seq samples.** 1) Quality control filtering thresholds used for each scRNA-seq and scATAC-seq sample (LNCaP, LNCaP-ENZ48, RES-A, and RES-B). 2) scRNA-seq sample metrics before quality control (as reported by 10x Genomics Cell Ranger) and after quality control using the Seurat package. 3) scATAC-seq sample metrics before quality control (as reported by 10x Genomics Cell Ranger ATAC) and after quality control using the Signac package.
