# Supplementary material for: Single-cell ATAC and RNA sequencing reveal pre-existing and persistent cells associated with prostate cancer relapse
Source: Nat Commun. 2021 Sep 6;12:5307. doi: 10.1038/s41467-021-25624-1 (PMC8421417; doi:10.1038/s41467-021-25624-1)
Supplement: Supplementary file 8 — Reporting Summary [file 41467_2021_25624_MOESM8_ESM.pdf]

nature research

Corresponding author(s): Alfonso Urbanucci and Matti Nykter

Last updated by author(s): Aug 16, 2021

Reporting Summary

Nature Research wishes to improve the reproducibility of the work that we publish. This form provides structure for consistency and transparency in reporting. For further information on Nature Research policies, see our Editorial Policies and the Editorial Policy Checklist.

Statistics

For all statistical analyses, confirm that the following items are present in the figure legend, table legend, main text, or Methods section.

n/a	Confirmed	
☐	☒	The exact sample size (*n*) for each experimental group/condition, given as a discrete number and unit of measurement
☒	☐	A statement on whether measurements were taken from distinct samples or whether the same sample was measured repeatedly
☐	☒	The statistical test(s) used AND whether they are one- or two-sided<br>*Only common tests should be described solely by name; describe more complex techniques in the Methods section.*
☒	☐	A description of all covariates tested
☐	☒	A description of any assumptions or corrections, such as tests of normality and adjustment for multiple comparisons
☐	☒	A full description of the statistical parameters including central tendency (e.g. means) or other basic estimates (e.g. regression coefficient) AND variation (e.g. standard deviation) or associated estimates of uncertainty (e.g. confidence intervals)
☐	☒	For null hypothesis testing, the test statistic (e.g. *F*, *t*, *r*) with confidence intervals, effect sizes, degrees of freedom and *P* value noted<br>*Give P values as exact values whenever suitable.*
☒	☐	For Bayesian analysis, information on the choice of priors and Markov chain Monte Carlo settings
☒	☐	For hierarchical and complex designs, identification of the appropriate level for tests and full reporting of outcomes
☐	☒	Estimates of effect sizes (e.g. Cohen's *d*, Pearson's *r*), indicating how they were calculated

Our web collection on statistics for biologists contains articles on many of the points above.

Software and code

Policy information about availability of computer code

Data collection	No code was used for data collection.
Data analysis	The following tools and software were used to perform the analyses: 10x Genomics Cell Ranger (version 3.0.2), 10x Genomics Cell Ranger ATAC (version 1.1.0), 10x Genomics Space Ranger (version 1.2.0), Seurat R package (version 3.2.0), Signac R package (version 0.2.5), Drop-seq tools (version 2.3.0), sctransform R package (version 0.3.1), fastMNN / batchelor R package (version 1.2.4), harmony R package (version 1.0), MAST R package (version 1.12.0), GSVA R package (version 1.34.0), fgsea R package (version 1.14.0), scVelo Python package (version 0.2.2), cytoTRACE R package (version 0.3.3), ggradar R package (version 0.2), TFBSTools R package (version 1.26.0), BSgenome.Hsapiens.UCSC.hg38 R package (version 1.4.1), clustree R package (version 0.4.3), survival R package (version 3.2-3), survminer R package (version 0.4.8), bwa (version 0.7.8-r455), Picard (versions 1.118 and 2.18.22), MACS2 (version 2.1.0), MSPC (version 4.0.2), DiffBind (version 2.14.0), featureCounts (version 1.6.2), STAR (versions 2.5.4b and 2.7.3a), JASPAR2018 R package (version 1.1.1), loompy (version 3.0.0), ReactomePA R package (version 1.30.0).

For manuscripts utilizing custom algorithms or software that are central to the research but not yet described in published literature, software must be made available to editors and reviewers. We strongly encourage code deposition in a community repository (e.g. GitHub). See the Nature Research guidelines for submitting code & software for further information.

Data

Policy information about availability of data

All manuscripts must include a data availability statement. This statement should provide the following information, where applicable:

- Accession codes, unique identifiers, or web links for publicly available datasets
- A list of figures that have associated raw data
- A description of any restrictions on data availability

The single-cell RNA, single-cell ATAC, FAIRE-seq, and RNA-seq data generated in this study have been deposited in the Gene Expression Omnibus (GEO) archive under accessions GSE168669 [https://www.ncbi.nlm.nih.gov/geo/query/acc.cgi?acc=GSE168669] and GSE168733 [https://www.ncbi.nlm.nih.gov/geo/query/acc.cgi?acc=GSE168733]. The spatial transcriptomics data are available at the European Genome-Phenome Archive (EGA) under identifier EGAS00001000526 [https://ega-archive.org/studies/EGAS00001000526]. Other publicly available datasets utilized but not generated in this study were: AR and c-MYC binding site maps (GEO archive: GSE73994 [https://www.ncbi.nlm.nih.gov/geo/query/acc.cgi?acc=GSE73994]), bulk RNA-sequencing of LNCaP samples analyzed using single-cell methods in this study (GEO archive: GSE130534 [https://www.ncbi.nlm.nih.gov/geo/query/acc.cgi?acc=GSE130534]), xenografts of AR-positive/NE-negative and AR-negative/NE-positive CRPC tumors (GEO archive: GSE124704 [https://www.ncbi.nlm.nih.gov/geo/query/acc.cgi?acc=GSE124704] and GSE126078 [https://www.ncbi.nlm.nih.gov/geo/query/acc.cgi?acc=GSE126078]), single-cell RNA-seq of 13 treatment-naive prostate tumor samples (GEO archive: GSE141445 [https://www.ncbi.nlm.nih.gov/geo/query/acc.cgi?acc=GSE141445]), LNCaP xenograft models of CRPC (Supplementary File 1 in King et al., 2017 [https://doi.org/10.18632/oncotarget.22560]), patient RNA-sequencing from enzalutamide responders and non-responders [https://doi.org/10.1073/pnas.1922207117], RNA-sequencing from SU2C CRPC patient samples [https://doi.org/10.1073/pnas.1902651116], RNA-sequencing from SU2C West Coast DT patient samples [https://doi.org/10.1016/j.cell.2018.06.039], spatial transcriptomics data from prostate tissue sections 1.2, 2.4, and 3.3 [https://doi.org/10.1038/s41467-018-04724-5], TCGA-PRAD RNA-seq [https://portal.gdc.cancer.gov/], and ICGC-EOPC RNA-seq [https://doi.org/10.1016/j.ccell.2018.10.016]. Databases utilized in the study were the Molecular Signatures Database (MSigDB) v7.0 (hallmark gene sets, http://www.gsea-msigdb.org/gsea/msigdb/index.jsp), the GTRD database v18.06 (https://gtrd.biouml.org/), and the Housekeeping and Reference Transcript Atlas v1.0 (http://www.housekeeping.unicamp.br/).
The remaining data are available within the Article, Supplementary Information, or Source Data file. Unique biological materials are available from the corresponding authors upon reasonable request. Source data are provided with this paper.

Field-specific reporting

Please select the one below that is the best fit for your research. If you are not sure, read the appropriate sections before making your selection.

- [x] Life sciences
- [ ] Behavioural & social sciences
- [ ] Ecological, evolutionary & environmental sciences

For a reference copy of the document with all sections, see nature.com/documents/nr-reporting-summary-flat.pdf

Life sciences study design

All studies must disclose on these points even when the disclosure is negative.

Sample size	We utilized four single-cell samples from LNCaP that were chosen to represent different time points during the development of resistance to enzalutamide in the cells. One control LNCaP sample was not treated with enzalutamide, one sample was treated with enzalutamide for 48 hours to represent short-term response to the drug, and two samples were resistant to enzalutamide. Three additional single-cell samples (LNCaP treated with enzalutamide for 168 hours, untreated VCaP, and VCaP treated with enzalutamide for 48 hours) and six LNCaP FAIRE-seq samples were used to supplement our understanding of cell states along the path to enzalutamide resistance. Five bulk RNA-seq samples from VCaP were similarly chosen to represent different states of resistance to androgens, bicalutamide, and/or enzalutamide. Single cell technology allow measurement of signal from multiple cells at the same time, and we aimed at obtaining information from similar amounts of cells per sample. Therefore, we did not calculate sample size for the single cell samples and no statistical methods were used to determine sample size.
Data exclusions	No data were excluded from the analysis.
Replication	The LNCaP FAIRE-seq and VCaP bulk RNA-seq experiments were performed with 3 biological replicates. All attempts at replication were successful. No replicates were performed for the single-cell samples as the sequencing of each sample resulted in an expected number of cells for the 10x Genomics and Drop-seq platforms.
Randomization	This study is not interventional and no groups were allocated, so randomization was not necessary.
Blinding	This study is not interventional and no groups were allocated, so blinding was not necessary.

Reporting for specific materials, systems and methods

We require information from authors about some types of materials, experimental systems and methods used in many studies. Here, indicate whether each material, system or method listed is relevant to your study. If you are not sure if a list item applies to your research, read the appropriate section before selecting a response.

Materials & experimental systems

n/a	Involved in the study
☒	☐ Antibodies
☐	☒ Eukaryotic cell lines
☒	☐ Palaeontology and archaeology
☒	☐ Animals and other organisms
☒	☐ Human research participants
☒	☐ Clinical data
☒	☐ Dual use research of concern

Methods

n/a	Involved in the study
☒	☐ ChIP-seq
☒	☐ Flow cytometry
☒	☐ MRI-based neuroimaging

Eukaryotic cell lines

Policy information about cell lines

Cell line source(s)	The LNCaP and VCaP cell lines were obtained from American Type Culture Collection (ATCC; LGC Standards). The VCaP cell line (passage (p.) 15.) used for deriving drug-resistant sublines was a gift from Dr. Tapio Visakorpi, Tampere University, Finland.
Authentication	All LNCaP and VCaP cell lines were authenticated periodically (HPA cultures or Eurofins).
Mycoplasma contamination	All LNCaP and VCaP cell lines were tested monthly for mycoplasma and no mycoplasma contamination was detected.
Commonly misidentified lines (See ICLAC register)	None
